# Does post acute care reduce the mortality of octogenarian and nonagenarian patients undergoing hip fracture surgery?

**DOI:** 10.1186/s12877-024-04936-z

**Published:** 2024-04-08

**Authors:** Yu-Wei Chiang, Yu-Jun Chang, Hui-Jen Huang, Cheng-Pu Hsieh, Yueh-Hsiu Lu

**Affiliations:** 1 Department of Orthopedics, Changhua Christian Hospital, No. 135, Nanxiao St., Changua City, Changhua County 500054 Taiwan R.O.C.; 2 Big Data Center, Changhua Christian Hospital, No. 135, Nanxiao St., Changua City, Changhua County 500054 Taiwan R.O.C.; 3 Department of Nursing, Changhua Christian Hospital, No. 135, Nanxiao St., Changua City, Changhua County 500054 Taiwan R.O.C.; 4 Orthopedics & Sports Medicine Laboratory, Changhua Christian Hospital, No. 135, Nanxiao St., Changua City, Changhua County 500054 Taiwan R.O.C.; 5 Department of Post-Baccalaureate Medicine, National Chung Hsing University, No. 145 Xingda Rd., South District, Taichung, 40227 Taiwan R.O.C.; 6 Institute of Biomedical Sciences, National Chung Hsing University, No. 145 Xingda Rd., South District, Taichung, 40227 Taiwan R.O.C.

**Keywords:** Hip fracture, Fragile fracture, Osteoporosis, Post-acute-care, Mortality

## Abstract

**Background:**

With the increasing number of elderly individuals worldwide, a greater number of people aged 80 years and older sustain fragility fracture due to osteopenia and osteoporosis.

**Methods:**

This retrospective study included 158 older adults, with a median age of 85 (range: 80–99) years, who sustained hip fragility fracture and who underwent surgery. The patients were divided into two groups, one including patients who joined the post-acute care (PAC) program after surgery and another comprising patients who did not. The mortality, complication, comorbidity, re-fracture, secondary fracture, and readmission rates and functional status (based on the Barthel index score, numerical rating scale score, and Harris Hip Scale score) between the two groups were compared.

**Results:**

The patients who presented with fragility hip fracture and who joined the PAC rehabilitation program after the surgery had a lower rate of mortality, readmission rate, fracture (re-fracture and secondary fracture), and complications associated with fragility fracture, such as urinary tract infection, cerebrovascular accident, and pneumonia (acute coronary syndrome, out-of-hospital cardiac arrest, or in-hospital cardiac arrest.

**Conclusions:**

PAC is associated with a lower rate of mortality and complications such as urinary tract infection, bed sore, and pneumonia in octogenarian and nonagenarian patients with hip fragility fracture.

## Introduction

There is an increasing number of elderly individuals worldwide, particularly in developed countries. The number of people with fragility fractures caused by conditions such as osteopenia and osteoporosis are rising. Fragility fractures are associated with significant morbidity and mortality [1]. Some studies have revealed that Asians commonly have an increased rate of hip fracture [2]. Various studies have reported that the 1-year cumulative mortality rates after hip fractures range from 20 to 40% [3–5]. The primary contributing factors to this high mortality rate are decreased independence, prolonged bedridden periods, and poor functional status, which can lead to the development of additional comorbidities and a diminished quality of life. Among all types of fragility fractures, such as those occurring in the hip, spine, and distal forearm, hip fractures are particularly debilitating [6, 7]. The post-acute care (PAC) program can be an important transitional phase between hospital admission and home care. The essence of the PAC program is to seize the golden period of rehabilitation after surgery and can improve activities of daily living and early mobilization, which are associated with better outcomes [8]. Numerous studies have shown that PAC significantly enhances functional status [9–11], reduces the risk of readmission [12], and decreases the mortality rate [13]. However, there is limited quantity of study that examined the efficacy of PAC for patients aged 80 years and over. Therefore, we focused on this patient population in the current study. In addition, we aimed to evaluate the efficacy of PAC for fragility fractures by examining its impact on reducing re-fracture rates and the need for subsequent surgeries. Further, the effect of increased rehabilitation intensity on alleviating complication, comorbidity, and even mortality in patients with fragility hip fracture was investigated. By analyzing these aspects, we hope to gain insights into the potential benefits of fragility fracture PAC in improving outcomes and overall well-being in this particular patient group.

## Materials and methods

Orthopedic and rehabilitation physicians assess whether patients are suitable for enrollment in the PAC program. This article focuses on evaluating the effectiveness of PAC for octogenarian and nonagenarian patients with hip fracture after surgery. The inclusion criteria were as follows: (1) patients aged > 80 years; (2) those diagnosed with hip fractures, including femoral neck fractures, intertrochanteric fractures, and subtrochanteric fractures; (3) those who underwent hip surgery such as hip hemiarthroplasty, cephalomedullary nail, dynamic hip screw, or cannulated screw fixation; (4) those with stable vital signs, including blood pressure, heart rate, body temperature, respiratory rate, and blood oxygen saturation in the past 72 h; (5) those with stabilized or controllable complications; (6) those with good cognitive function with learning ability; (7) those have sufficient physical strength to undergo at least 1 h or more of active rehabilitation therapy daily. The exclusion criteria were as follows: (1) patients with an expected lifetime of < 6 months; (2) those requiring ventilator support; (3) those requiring consecutive treatments such as chemotherapy, radiotherapy, and other surgical procedures; (4) those with cognitive disorders affecting consciousness; (5) those with spine fractures accompanied with spinal cord injuries; and (6) those who were lost to follow-up. Once a patient meets the aforementioned criterias, we inquire with the primary caregiver and family members their willingness to participate in PAC. When the patient experiences cognitive impairment or delirium, our priority is to identify the underlying causes and initiate appropriate treatment. If the patient regains cognitive function, they can then enter the PAC program. If the patient's symptoms persist without improvement, they will not be eligible for PAC. In patients participating in the PAC rehabilitation program, a more intensive rehabilitation regimen was implemented, comprising two sessions per day, with each session lasting approximately 50 min. The schedule of these sessions was individualized to each patient’s rehabilitation potential and individual needs. For patients who do not accept PAC, we still arrange rehabilitation with a physical therapist, but the intensity and frequency may not be as high. Additionally, there won't be a case manager, who customizes the rehabilitation plan for the patient, makes arrangements post-discharge, provides care for the patient through phone calls or visiting their home after discharge. Family members of the patient must proactively seek suitable nursing facilities or make their own arrangements for post-discharge preparation upon returning home. The study protocol was conducted according to the guidelines of the Declaration of Helsinki, and the individual informed consent for this retrospective analysis was waived by the Institutional Review Board of Changhua Christian Hospital (No. 210611). Data collection was performed between January 2020 and December 2021, during which 158 eligible patients were included in the analysis. The clinical characteristics of the participants, such as age, sex, Barthel index, numerical rating scale score, Harris Hip Scale score, bone mineral density, treatment for osteoporosis, preoperative and postoperative radiography results, and participation in the PAC program, were reviewed and recorded. The outcome analysis encompassed several measures, including re-fracture rate (occurring at the same site as the primary fracture), duration of re-fracture, second fracture rate (occurring at a different site compared with the primary fracture), duration of second fracture, re-operation rate and duration, readmission rate and duration, and improvement in Barthel index, numerical rating scale score, and Harris Hip Scale score. Ultimately, morbidity and mortality outcomes were studied.

### Statistical analysis

In total, 158 participants were enrolled in this study, and their demographic characteristics and baseline data were recorded comprehensively. Before and after undergoing the PAC rehabilitation program, the participants’ functional abilities were assessed using the Barthel index, and pain levels were measured using the numerical rating scale. Hip function was evaluated using the Harris Hip Scale score. To determine the significance of the changes observed, the Wilcoxon signed-rank test was utilized. Statistical methods were used to examine the association between PAC and various outcomes such as mortality, re-fracture, complications, and readmission rates. The chi-square test or the Fisher’s exact test was used, as appropriate, to analyze data and determine any significant associations. In addition, the log-rank test was utilized to estimate the hazard ratio, particularly focusing on the association between re-fracture rates and variables such as bone marrow density, cardiovascular disease, and diabetes. Furthermore, the Cox proportional-hazards regression model was used to analyze the association between mortality and various comorbidities or complications, including pneumonia, septic shock, and cancer, and postoperative osteoporosis treatment. These statistical analyses were conducted to explore the potential impact of PAC on mortality, re-fracture, complication, and readmission rates among individuals with hip fragility fractures. Furthermore, the influence of factors such as bone marrow density, cardiovascular disease, diabetes, and various complications on the risk of re-fracture and mortality was investigated. These findings can contribute to a better understanding on the role of PAC and its potential benefits in enhancing the outcomes and overall prognosis of patients with fragility fractures.

## Results

**Fig. 1:**
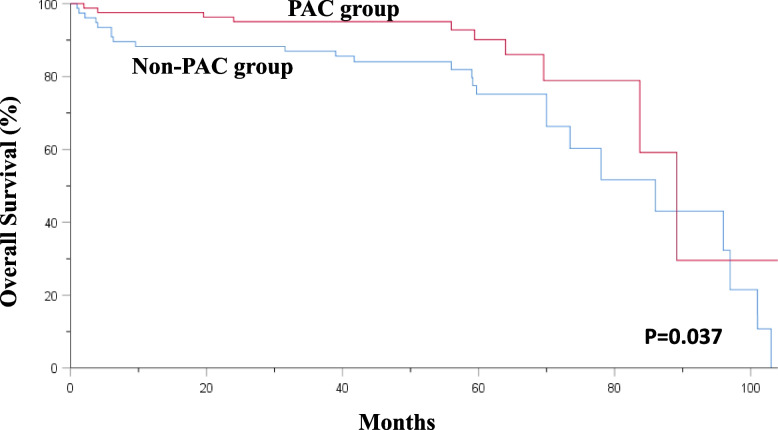
The patient’s mortality rate in PAC and non-PAC group

**Fig. 2:**
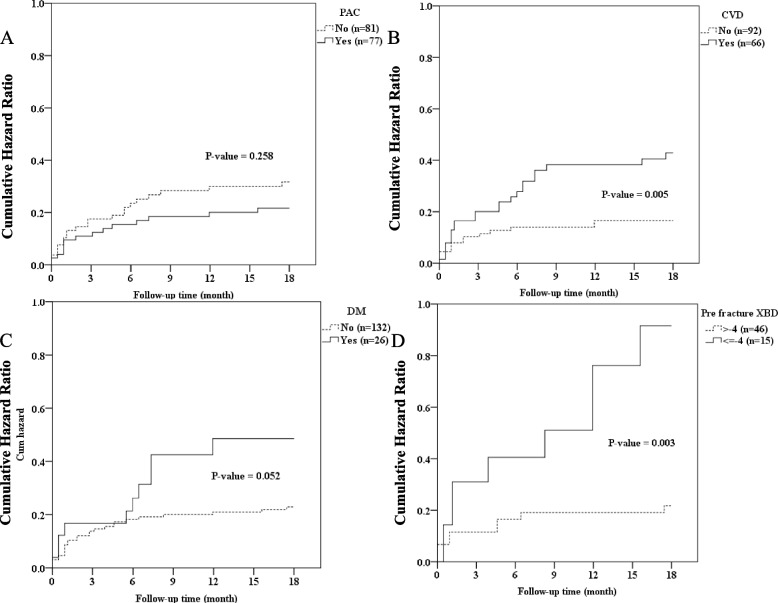
Kaplan–Meier estimates the cumulative incidence of re-fracture. **A** PAC group versus non-PAC group (**B**) patient with CVD or not (**C**) patient with diabetes mellitus or not (**D**) patient with bone marrow density ≤ -4 and > -4 before fracture

In total, 158 patients were included in this study. Table 1 shows the baseline characteristics of the participants. Among the participants, 50 were men and 108 women, with a median age of 85 (range: 80–99) years. The distribution of fractures among the patients was as follows: 15 (9.5%) patients had Garden type I–II femoral neck fracture; 44 (27.8%), Garden type III–IV femoral neck fracture; 37 (23.4%), stable intertrochanteric fracture; 57 (36.1%), unstable intertrochanteric fracture; 1 (0.6%), basal neck fracture; 3 (1.9%), femoral neck fracture treated with cannulated screw fixation requiring revision due to loss reduction; and 1 (0.6%), subtrochanteric fracture. Regarding the surgical interventions, 14 (8.9%) patients underwent cannulated screw fixation; 48 (30.4%), hip hemiarthroplasty; 56 (35.4%), short cephalomedullary nail fixation; 35 (22.2%), long cephalomedullary nail fixation; and 5 (3.2%), dynamic hip screw fixation. The patients were divided into two groups, one comprising patients who participated in the PAC rehabilitation program and the other including patients who did not. These baseline characteristics could provide a comprehensive overview of the study population and facilitate the analysis of the impact of the PAC rehabilitation program on various outcomes among patients with fragility hip fractures. In this study, all patients presented with hip fragility fractures. Further, 77 (48.7%) patients joined the PAC rehabilitation program, and 81 (51.3%) did not. In the non-PAC group, 25 patients died during follow-up and the mortality rate was 30.9%. In contrast, 10 patients died during follow-up and the mortality rate was 13% in the PAC group. The patients who received PAC rehabilitation had a significant lower mortality rate than those who did not ( *P* = 0.03) (Fig. 1, Table 2). The median times to mortality in the non-PAC and PAC groups were 1.26 and 5.51 months, respectively (Table 3). In addition, patients who participated in the PAC rehabilitation program had a lower readmission rate under 18 months of follow-up (39% vs.49.4%, *P* = 0.187) (Table 4). Furthermore, the PAC group also had a lower re-fracture rate (2.6% vs. 4.9%, *P* = 0.682) (Table 4) and second fracture rate (18.2% vs. 24.7%, *P* = 0.3) (Table 4). Re-fracture was defined as another fracture episode occurring in the same site and second fracture was defined as a fracture episode in an area different from the initial fracture site. Because of underlying osteoporotic status, patients in our study population tend to sustain fragility fractures again and receive second or even third operation. The incidence of re-fracture plus second fracture events were higher in PAC group than non-PAC group (27.2% vs. 19.5%, *P* = 0.258) (Table 5, Fig. 2A).

**Table 1:** Patients’ clinical characteristics and operation methods

Characteristics		Total (*n* = 158)		No PAC (*n* = 81)		PAC (*n* = 77)	
		N	%	N	%	N	%
Gender	Female	108	68.4	54	66.7	54	70.1
	Male	50	31.6	27	33.3	23	29.9
Diagnosis	Femoral neck fracture, Garden type I ~ II	15	9.5	10	12.3	5	6.5
	Femoral neck fracture, Garden type III ~ IV	44	27.8	21	25.9	23	29.9
	Stable ITF	37	23.4	18	22.2	19	24.7
	Unstable ITF	57	36.1	30	37.0	27	35.1
	Basal neck fracture	1	0.6	1	1.2	0	0.0
	Femoral neck fr s/p cannulated screw fixation and loss reduction	3	1.9	1	1.2	2	2.6
	Subtrochanteric fracture	1	0.6	0	0.0	1	1.3
Diagnosis	Femoral neck fracture	59	37.3	31	38.3	28	36.4
	ITF	94	59.5	48	59.3	46	59.7
	Others	5	3.2	2	2.5	3	3.9
Total OP time	1	131	82.9	67	82.7	64	83.1
	2	23	14.6	11	13.6	12	15.6
	3	4	2.5	3	3.7	1	1.3
OP method	Cannulated screw	14	8.9	10	12.3	4	5.2
	Hemiarthroplasty	48	30.4	22	27.2	26	33.8
	Short CM nail	56	35.4	29	35.8	27	35.1
	Long CM nail	35	22.2	16	19.8	19	24.7
	DHS	5	3.2	4	4.9	1	1.3
Second OP	No	132	83.5	68	84.0	64	83.1
	Yes	26	16.5	13	16.0	13	16.9
Third OP	No	154	97.5	78	96.3	76	98.7
	Yes	4	2.5	3	3.7	1	1.3
Pre-Osteoporosis Tx	No	140	88.6	71	87.7	69	89.6
	Yes	18	11.4	10	12.3	8	10.4

*Abbreviations: PAC* post-acute care, *ITF* intertrochanteric fracture, *fr* fracture, *OP* operation, *DHS* dynamic hip screw, *Tx* treatment, *CM* cephalomedullary

**Table 2:** The patients mortality rate in PAC group and non-PAC group

	Patient number	Death	%	Mean Survival Time (months)	SE	95% C.I	*P*-value
Non-PAC	81	25	30.9	15.5	0.6	14.2–16.8	0.037
PAC	77	10	13	16.8	0.5	15.9–17.7	
Overall	158	35	22.2	16.1	0.4	15.3–16.9

**Table 3:** The duration of mortality in PAC group and non-PAC group

	Death number	Mean (M)	SD (M)	Q1 (M)	Median (M)	Q3 (M)	Min (M)	Max (M)
Non-PAC	25	3.46	4.86	0.92	1.26	6.43	0.23	17.90
PAC	10	7.88	7.02	0.92	5.51	12.85	0.46	17.44

*Abbreviation*: *M* months

**Table 4:** Patients’ clinical outcomes after operation

Outcomes		Total (*n* = 158)		No PAC (*n* = 81)		PAC (*n* = 77)	
		N	%	N	%	N	%
Re-fracture	No	152	96.2	77	95.1	75	97.4
	Yes	6	3.8	4	4.9	2	2.6
Second fracture	No	124	78.5	61	75.3	63	81.8
	Yes	34	21.5	20	24.7	14	18.2
Mortality	No	123	77.8	56	69.1	67	87
	Yes	35	22.2	25	30.9	10	13.0
Re-admission	No	88	55.7	41	50.6	47	61.0
	Yes	70	44.3	40	49.4	30	39.0

**Table 5:** Cumulative incidence of re-fracture different groups of patients

		Patient number	Re-fracture& Second fracture	%	Mean Survival Time (months)	SE	95% C.I	*P*-value
PAC	Non-PAC	81	24	29.6	14.2	0.7	12.7–15.6	0.258
	PAC	77	16	20.8	15.3	0.7	13.9–16.6	
	Overall	158	37	25.6	14.7	0.5	13.7–15.7	
CVD history	No	92	14	15.2	15.7	0.6	14.6–16.9	**0.005**
	Yes	66	23	34.8	13.3	0.9	11.6–15.0	
	Overall	158	37	23.4	14.7	0.5	13.7–15.7	
DM history	No	132	27	20.5	15.1	0.5	14.0–16.1	**0.052**
	Yes	26	10	38.5	12.9	1.4	10.2–15.5	
	Overall	158	37	23.4	14.7	0.5	13.7–15.7	
XBD before fracture	> -4	46	9	19.6	15.2	0.9	13.5–17.0	**0.003**
	< = -4	15	9	60.0	10.9	1.9	7.2–14.5	
	Overall	61	18	29.5	14.2	0.9	12.5–15.8

In the PAC group, 16.9% and 1.3% of patients required a second and third operation, respectively. In the non-PAC group, 16.0% and 3.7% of patients required a second and third operation, respectively (Table 1). Among all fragility fractures, vertebral compression fracture was the most common reason for the need of an operation such as vertebroplasty and kyphoplasty.

This study evaluated the following complications associated with fragility fractures in the PAC group and the non-PAC group: urinary tract infection (6.5% vs. 11.1%, *P* = 0.439), cerebrovascular accident (1.3% vs.6.2%, *P* = 0.211), pneumonia (6.5% vs. 9.9%), acute coronary syndrome (0% vs. 2.5%), and out-of-hospital cardiac arrest or in-hospital cardiac arrest (0% vs. 3.7%) (Table 6). In our study, the PAC group had a significantly lower mortality rate and decreased rate of complications such as urinary tract infection, pneumonia, cerebrovascular accident, and acute coronary syndrome than the non-PAC group. However, these results except mortality rate did not significantly differ. The Barthel index, numerical rating scale, and Harris Hip Score were used to assess the general condition of the patients. The median Barthel index before PAC was 40. After receiving the PAC, the index improved to 45 (*P* < 0.001). The median Harris Hip Scale score improved from 32 to 39 (*P* = 0.133). The median NRS score was similar. However, there was a trend in score improvement (*P* = 0.052) after the PAC program (Table 7). Therefore, the PAC group had significant improvement in patients’ activities of daily living according to the Barthel index in the current study. Among all the patients, 140 (88.6%) presented with osteoporosis, which is defined as a bone density of 2.5 standard deviations below that of a young adult by the World Health Organization (WHO), and the remaining presented with osteopenia. The PAC group had a higher wellness level to receive osteoporotic medication than the non-PAC group (58.4% vs. 41.6%). The patient who received osteoporotic medications had a lower re-fracture rate (93.2% vs. 6.8%, *P* = 0.034), second fracture rate (73% vs.27%, *P* = 0.048), and lower mortality rate (7% vs. 93%, *P* = 0.002). In conclusion, patients with osteoporotic treatment had significantly lower re-fracture rate, secondary fracture rate, and mortality rate in the PAC group.

**Table 6:** Complication of fragility in PAC group and non-PAC group

Complications		Total		Non-PAC group		PAC group	
		N	%	N	%	N	%
CVA	No	152	96.2	76	93.8	76	98.7
	Yes	6	3.8	5	6.2	1	1.3
UTI	No	144	91.1	72	88.9	72	93.5
	Yes	14	8.9	9	11.1	5	6.5
Pneumonia	No	145	91.8	73	90.1	72	93.5
	Yes	13	8.2	8	9.9	5	6.5
ACS	No	156	98.7	79	97.5	77	100
	Yes	2	1.2	2	2.5	0	0
OHCA/IHCA	No	155	98.1	78	96.3	77	100
	Yes	3	1.9	3	3.7	0	0

**Table 7:** Evaluation the efficacy of Post-acute-care

		Score			Score change patient number (N)			*P*-value
		Median	Q_1_	Q_3_	Decreased	Increased	Unchange	
Barthel index (score 0–100)	Pre-PAC	40	40	45				
	Post- PAC	45	40	55	5	20	32	0.001
NRS (score 0–10)	Pre- PAC	4	3	5				
	Post- PAC	4	2	4	23	14	18	0.052
Harris hip score (score 0–100)	Pre- PAC	32	29	42				
	Post- PAC	39	28	49	21	33	1	0.133

## Discussion

Fragility hip fracture, which may cause a significant burden on families and the society, is a major concern among elderly individuals [14]. A cohort study has shown that the annual mortality rate of hip fracture has decreased from 18 to 14% from 1999 to 2009 in Taiwan [15]. A 10-year study on mortality and mobility in patients with hip fracture in Japan revealed that the survival rate after hip fracture event decreased and the mortality risk remained high for 10 years. This risk was approximately twice as that in the general population, even at 10 years after fracture. For all elderly patients with hip fractures postoperatively, we routinely assess whether these patients are suitable for participation in the PAC program. The PAC program is different from the general rehabilitation arrangement. Once the patient joined the PAC program, the specialized case managers would provide a tailored approach to managing the unique needs of individuals with fragile bone fractures. Various healthcare professionals would be involved in the PAC program such as orthopedic surgeons, physiatrists, physiotherapists, and other specialists. Our goal includes comprehensive assessment of the patient's condition, facilitating a more personalized and effective treatment plan. In our PAC program, in addition to the acute postoperative ward stay, patients participating in the PAC program extend their hospitalization by three more weeks. We arranged rehabilitation sessions twice in a day, and it took one hour each time. Relative high-intensity rehabilitation and intensive training made patients restore mobility, strength, and functionality, it also reduced the risk of complications and improved the overall quality of life for the patients. After the patient is transferred to the Nursing Organization or back to their home, the PAC teams conduct visits to the transferred cases, providing care and support.

A retrospective study analyzing the postoperative mortality and morbidity in octogenarians and nonagenarians with hip fracture indicated that gender and age are risk factors for the mortality. Women and the elderly are more likely to suffer from complications after operation. This study also reported the mortality rate after hip fracture to be about 20.4% [16]. Another recent retrospective study aimed to evaluate the nonagenarian hip fractures mortality after hip surgery. Their 30-day mortality and 1-year mortality rate were 3.9% and 38.1%, respectively [17]. Compared to our study with the mortality of 13% in the PAC group. Joining the PAC plays an important role in decreasing the mortality rate [18]. According to a previous study with a 1-year follow-up, 40% of patients with hip fracture could not walk independently, 60% required assistance, and 33% were totally dependent or had long-term living experiences in a nursing home [19, 20]. A systematic review revealed that the cost of hip fracture-related hospitalization was $10,075, and the health and social care cost for 12 months was $43,669, with inpatient costs being their major driver [21]. According to our result, the PAC is associated with a decreased mortality rate in patients with fragility hip fracture and a low complication rate (such as urinary tract infection and pneumonia) in patients with hip fracture. One prospective study revealed that approximately 8.16% and 1.29% of patients who are bedridden developed pneumonia and urinary tract infection, respectively. These complications were significantly associated with mortality and reduced quality of life [22]. The mortality of patients with pneumonia due to prolonged bedridden has significantly increased compared with that of patients without pneumonia (hazard ratio [HR]: 3.7, 95% confidence interval [CI]: 1.379–10.047, *P* = 0.009).

The patients with re-fracture (at the same site and another fracture site) have a higher mortality rate than those without re-fracture (2.7% vs. 18.2%, *P* = 0.019). Other factors were associated with mortality. Bokshan et al. found that a 48-h surgical delay would increase the mortality for the relatively healthier patient; but delay surgery can be a protective factor for the relatively illness patients. Both the octogenarians and nonagenarians had higher mortality rate, and returned to as the normal population after 4 years and 5 years postoperative, respectively [23]. Post acute care is actually protective against mortality for octogenarians and nonagenarians following hip fracture surgery, by bridging the gap between the hip fracture patient and normal population. The PAC reduced immobilized time and bed rest time, which lead to less comorbidity such as pneumonia, urinary tract infection and bed sore. As a result, joining PAC improves the mortality rate and relative complications. The early rehabilitation breaks the cycle of virchow’s triad and lowers the incidence of thromboembolic events.

A recent study revealed that both anti-resorptive and anabolic medications may be beneficial for bone healing progress [24]. In our study, the patient who received osteoporotic treatment had a reduced rate of re-fracture, second fracture, and mortality (HR: 0.263, 95% CI: 0.098–0.709, *P* = 0.008). The osteoporotic medication could also have decreased the mortality rate (Table 8).The readmission rate was positively correlated with mortality (HR: 3.3, 95% CI: 1.176–9.261, *P* = 0.023) (Table 8). Readmission had several causes, which include re-fracture and immobility complications such as pneumonia, urinary tract infection, and deep vein thrombosis. PAC has a positive effect on delirium. Also, transdisciplinary care improves health literacy and health concepts not only in patients but their family. Their family or caregivers may pay more attention to minor symptoms. Early detection of minor symptoms prevents major problems. Therefore, PAC lowered emergency department visits and readmission rate [25, 26]. Data on the patients’ underlying illness were also reviewed. Results showed that cardiovascular disease (Fig. 2B, Table 3), diabetes mellitus (Fig. 2C, Table 3), and low bone marrow density were associated with a higher re-fracture rate (Fig. 2D, Table 3). The Cox proportional-hazards regression model was used to analyze mortality. Results revealed that several factors such as hematologic disease, cancer history, pneumonia episode during hospitalization, septic shock, and readmission were associated with mortality (Table 8).

**Table 8:** Cox proportional-hazards regression analysis of mortality

Characteristic			Dead		Bivariable analysis (crude)			Multivariable analysis (adjusted)		
		Total	N	%	HR	95% CI	*P*-value	HR	95% CI	*P*-value
Gender	Female	108	12	11.1	1.00					
	Male	50	11	22.0	2.15	0.95–4.87	0.067			
Hematologic disease	No	154	19	12.3	1.00					
	Yes	4	4	100	23.2	7.21–74.67	< 0.001			
Cancer history	No	143	15	10.5	1.00			1.00		
	Yes	15	8	53.3	6.23	2.64–14.72	< 0.001	13.71	3.86–48.71	< 0.001
PAC	No	81	14	17.3	1.00					
	Yes	77	9	11.7	0.64	0.28–1.49	0.303			
OP method	Cannulated screw	14	1	7.1	1.00					
	Hemiarthroplasty	48	9	18.8	2.84	0.36–22.39	0.323			
	Short CM nail	56	10	17.9	2.63	0.34–20.52	0.357			
	Long CM nail	35	1	2.9	0.39	0.02–6.25	0.507			
	DHS	5	2	40.0	6.97	0.63–76.95	0.113			
Complicated with pneumonia during hospitalization	No	145	18	12.4	1.00					
	Yes	13	5	38.5	3.72	1.38–10.04	0.009			
Complicated with septic shock during hospitalization	No	155	20	12.9	1.00			1.00		
	Yes	3	3	100	25.9	7.14–94.01	< 0.001	103.02	20.34–521.75	< 0.001
Received Tx of osteoporosis after OP	No	70	17	24.3	1.00			1.00		
	Yes	88	6	6.8	0.24	0.10–0.62	0.003	0.26	0.10–0.71	0.008
Re-admission	No	88	8	9.1	1.00			1.00		
	Yes	70	15	21.4	2.38	1.00–5.59	0.049	3.30	1.18–9.26	0.023
Re-admission due to pneumonia	No	145	16	11.0	1.00					
	Yes	13	7	53.8	5.93	2.43–14.48	< 0.001			

*Abbreviations*: *PAC* post-acute care, *DHS* dynamic hip screw, *Tx* treatment, *OP* operation, *CM* cephalomedullary

Along with the progression of the surgical technique, implant design, and preoperative management protocol, there is still lots of room for improvement in post-surgical care protocols. Previously, a patient was discharged if his/her general condition was stable and the total length of hospitalization was approximately 5–7 days. However, there is not enough time for the patient to receive a postoperative rehabilitation course and for the patient’s family to prepare rehabilitation equipment and environment. The National Health Insurance Agency developed the bundle payment system to improve the program’s quality and maintain its cost-effectiveness. At the medical center, the length of hospital stay after hip fracture surgery was limited to keep the surplus. Moreover, patients were transferred to the area or regional hospital for PAC and rehabilitation. The PAC program can be a transition period between admission and home care. Our hospital has different PAC programs such as stroke PAC [27], traumatic brain injury PAC [28], heart failure PAC [29], burn injury PAC [30], and/or fragility fracture PAC. A 5-year study has shown that patients who received the integrated Hip Fracture Care Pathway program can have better outcomes. That is, they have low complication and 30-day readmission rates, shorter length of hospitalization, and good functional status [31].

In our patient population, a pre-fracture bone marrow density T-score of <  − 4 could be associated with a higher incidence of another fracture event (Fig. 2). There is a consensus that the postoperative rehabilitation of hip fracture can improve the patient’s function and level of activities of daily living [32]. Our study had similar results, but not significant. Further, elderly patients were included in the current analysis. Results showed a limited progress without statistically significant differences, and it might be attributed to weak general condition and complex comorbidities. Therefore, we should individualize various PAC protocols for different age groups.

The current study had several limitations. First, the PAC program only started in 2019, and the number of participants was limited. Second, this was a retrospective study; thus, sampling bias might have existed. The follow-up time was only 1 and 1/2 years, and some studies showed no statistically significant difference. Third, there was no evaluation of complication severity. Some evaluation tools such as the numerical rating scale and Harris Hip Scale were subjective. Further, other patient characteristics such as muscle strength and hyperparathyroidism, which are associated with vitamin D deficiency, may affect mortality [33–35] and should be considered as a reference for predicting patient outcome. Lastly, there may be some selection bias, patients with extremely poor postoperative recovery, absence of rehabilitation willingness, or those dependent on life support systems cannot participate in the PAC program.

## Conclusions

The government and hospital policy advocate the efficacy of the PAC programs. In our study, patients in the PAC group had significantly lower mortality rate (*P* = 0.03). Although not statistically significant, lesser complications were observed, such as urinary tract infection, bed sore, and pneumonia in octogenarian and nonagenarian patients with hip fragility fracture in the PAC group. Our study supports the government policy that the PAC program improves outcomes in patient groups with fragility fracture.

## Data Availability

Data are available from Changhua Christian Hospital, Taiwan. The datasets used and analyzed during the current study are available from the corresponding author on reasonable request.

## Abbreviations

PAC: Post-acute care
CVA: Cerebrovascular accident
CI: Confidence interval
HR: Hazard ratio
DHS: Dynamic hip screw
Tx: Treatment
OP: Operation
CM: Cephalomedullary
ITF: Intertrochanteric fracture
fr: Fracture
